# Cytokinins and Abscisic Acid Act Antagonistically in the Regulation of the Bud Outgrowth Pattern by Light Intensity

**DOI:** 10.3389/fpls.2017.01724

**Published:** 2017-10-10

**Authors:** Adrien Corot, Hanaé Roman, Odile Douillet, Hervé Autret, Maria-Dolores Perez-Garcia, Sylvie Citerne, Jessica Bertheloot, Soulaiman Sakr, Nathalie Leduc, Sabine Demotes-Mainard

**Affiliations:** ^1^IRHS, Université d’Angers, INRA, Agrocampus-Ouest, SFR 4207 QUASAV, Beaucouzé, France; ^2^Institut Jean-Pierre Bourgin Centre de Versailles-Grignon (IJPB), INRA, Agro-ParisTech, CNRS, Versailles, France

**Keywords:** bud burst, PPFD, PAR, cytokinins, ABA, sugars, *Rosa hybrida* L., rose bush

## Abstract

Bud outgrowth is a key process in the elaboration of yield and visual quality in rose crops. Although light intensity is well known to affect bud outgrowth, little is known on the mechanisms involved in this regulation. The objective of this work was to test if the control of bud outgrowth pattern along the stem by photosynthetic photon flux density (PPFD) is mediated by sugars, cytokinins and/or abscisic acid in intact rose plants. Rooted cuttings of *Rosa hybrida* ‘Radrazz’ were grown in growth chambers under high PPFD (530 μmol m^-2^ s^-1^) until the floral bud visible stage. Plants were then either placed under low PPFD (90 μmol m^-2^ s^-1^) or maintained under high PPFD. Bud outgrowth inhibition by low PPFD was associated with lower cytokinin and sugar contents and a higher abscisic acid content in the stem. Interestingly, cytokinin supply to the stem restored bud outgrowth under low PPFD. On the other hand, abscisic acid supply inhibited outgrowth under high PPFD and antagonized bud outgrowth stimulation by cytokinins under low PPFD. In contrast, application of sugars did not restore bud outgrowth under low PPFD. These results suggest that PPFD regulation of bud outgrowth in rose involves a signaling pathway in which cytokinins and abscisic acid play antagonistic roles. Sugars can act as nutritional and signaling compounds and may be involved too, but do not appear as the main regulator of the response to PPFD.

## Introduction

Branching – or tillering for monocots – is a key process in the elaboration of crop yield and quality. Branching can indeed affect yield directly – through the number of fertile branches (Bredmose, 1993; Valério et al., 2009) – or indirectly by increasing the competitive ability of crops against weeds (Lemerle et al., 1996; Zhao et al., 2006) or by constraining pest infestation (Simon et al., 2012). In ornamental plants such as rose bush, plant architecture is derived from branching, and influences plant visual quality and thus consumers’ preferences (Boumaza et al., 2010; Garbez et al., 2015).

Bud outgrowth is a key event that determines whether a branch will develop or not. Not all axillary buds grow out along a stem because of correlative inhibitions between organs, which are processes whereby organs of a plant (apex, leaves, internodes, and other buds) can inhibit the outgrowth of a given bud (Brunel et al., 2002; Domagalska and Leyser, 2011). This produces a bud outgrowth pattern, which is a major component of plant architecture (Barthélémy and Caraglio, 2007). For example, under optimal growth conditions, the bud outgrowth pattern of a stem of the rose bush *Rosa hybrida* ‘Radrazz’ is acrotonic, meaning that apical buds grow out more frequently than median or basal buds (Demotes-Mainard et al., 2013b).

Bud outgrowth pattern can also be modified by changes in environmental factors, in particular light conditions. Photosynthetic photon flux density, red:far-red (R:FR), and blue/UV wavelengths have such effects (Leduc et al., 2014; Reddy et al., 2014; Demotes-Mainard et al., 2016; Huché-Thélier et al., 2016). The mechanisms behind light regulation of bud outgrowth are currently investigated. The effect of the R:FR ratio involves systemic auxin signaling, control of the branching repressor *BRC1* or of its homolog *TB1*, which in turn regulates ABA signaling and synthesis in the bud (Kebrom et al., 2006; González-Grandío et al., 2013, 2017; Reddy et al., 2013; Reddy and Finlayson, 2014; Holalu and Finlayson, 2017). Far less is known on how PPFD regulates bud outgrowth pattern and this is the question we address in this paper. Whether ABA, which inhibits bud outgrowth in various species (Chatfield et al., 2000; Cline and Oh, 2006; Reddy et al., 2013; Yao and Finlayson, 2015; González-Grandío et al., 2017), is also involved in regulating the response of bud outgrowth to PPFD remains for example unknown.

Sugars promote bud outgrowth (Bonhomme et al., 2009; Henry et al., 2011; Rabot et al., 2012; Mason et al., 2014; Barbier et al., 2015), but their role in the mediation of PPFD signal is also unclear. In rose, the inhibition of bud outgrowth by low PPFD is correlated to both a low sugar content in the stem and bud and to the down-regulation of a sucrose transporter gene (Furet et al., 2014). Yet, when in a decapitated defoliated rose exposed to darkness, sugars are supplied to the bud, this cannot suppress bud outgrowth inhibition, indicating that sugars are not the main limiting factor mediating outgrowth inhibition by darkness (Roman et al., 2016).

In Arabidopsis, Su et al. (2011) showed that reduced PPFD concomitantly inhibited rosette buds and up-regulated a repressor of CK signaling in the bud. The prominent role of CKs as initial targets of the light signal controlling bud outgrowth was demonstrated recently in rose (Roman et al., 2016). Dark inhibition of bud outgrowth involves rapid repression of the transcription of CK synthesis genes in the adjacent node and up-regulation of CK degradation genes in both the node and the bud, leading to a drop of the CK content in both organs. Conversely, exogenous supply of CKs to the bud or the adjacent node fully relieves inhibition of bud outgrowth by darkness. Exogenous CKs were shown to act through the regulation of a set of genes as rapidly and intensively as exposure to white light does. In particular, CKs in the node increase the sugar sink strength of the bud by up-regulating genes of sugar transport and metabolism (Roman et al., 2016).

These findings on the role of CKs were, however, obtained in response to white light or darkness in a simplified system consisting of the upper bud of a decapitated defoliated plant. In this system the bud is relieved from the apical dominance of the shoot tip and from the correlative inhibitions from other buds in an apical position, and from the influence of the leaves. The influence of the leaves is complex: mature leaves are a source of sugars for the whole plant, which promotes bud outgrowth, but young leaves can compete with buds for sugars, in addition mature leaves exert an inhibiting effect on the outgrowth of their adjacent bud (Zieslin and Halevy, 1976; Le Hir et al., 2006; Kebrom and Mullet, 2015). It remains unknown whether CKs are also key actors of the light control of bud outgrowth in whole plants. Moreover, their role was evidenced in response to extreme conditions (light vs. darkness), and whether CKs also mediate the response to PPFD variations within a range encountered under normal daylight conditions remains to be assessed. Considering the role of sugars and ABA in the control of bud outgrowth, interactions between CKs, ABA, and sugars in the control of branching pattern by PPFD need to be addressed.

We investigated these questions in rose plants subjected to high and low PPFD treatments that produce contrasted bud outgrowth patterns. We used intact plants, with buds submitted to the correlative inhibitions from other plant parts and determined the effect of PPFD on bud outgrowth pattern. Our previous studies in rose established that bud outgrowth is dependent on adjacent node resources, in particular sugars (Girault et al., 2010; Henry et al., 2011) and CKs (Roman et al., 2016) and that early light regulation of bud outgrowth takes place in the node adjacent to the bud (Roman et al., 2016). ABA which is synthesized in buds but also roots and leaves (Lacombe and Achard, 2016) circulates through the stem, and when applied to the stem ABA inhibits bud outgrowth (Arney and Mitchell, 1969; Chatfield et al., 2000; Cline and Oh, 2006). These results show the prominent role of the adjacent node on the control of the outgrowth of a bud. Therefore, to identify the early mechanisms of the control of bud outgrowth pattern by PPFD, we carried analyses on nodes. Sugar (starch, sucrose, glucose, and fructose) and hormone (CK, ABA, and IAA) contents were determined as well as the impact of exogenous supplies of sucrose, glucose, CKs, and ABA on bud outgrowth under two different light intensities. Correlations between these analyses and the establishment of a bud outgrowth pattern in response to PPFD are discussed.

## Materials and Methods

### Plant Material, Growth Conditions

Single-node cuttings of *R. hybrida* ‘Radrazz’ rose bushes were obtained as in Demotes-Mainard et al. (2013a). When the second leaf of the primary axis unfolded, the young plants were transferred to two identical growth chambers (Froids et Mesures, Beaucouzé, France). All lamps were metal halide lamps (HQI, OSRAM, München, Germany). The light spectrum in the growth chambers is presented in Supplementary Material 1, R:FR was 1.99 over the ranges (655–665 nm):(725–735 nm). Light spectrum was measured with a spectrophotometer (Avaspec-2048-6-RM, Avantes, Apeldoorn, The Netherlands). The plants were spaced 15 cm apart and surrounded by a border row. The photoperiod was 16h day/8h night, air temperature was 18°C day/17°C night, and humidity was 69%. The plants were sub-irrigated, and fertilized as in Demotes-Mainard et al. (2013a). The shoot apical meristem of the primary axis produced vegetative phytomers before differentiating into a terminal flower bud. The flower bud was first hidden within the unfolded young leaves until it became visible, and then referenced to as the FBV stage. It coincides in *R. hybrida* ‘Radrazz’ with the outgrowth of the first buds on the primary axis.

### Light Treatments

All plants were exposed to a to a PPFD of 529 ± 6 μmol m^-2^ s^-1^ starting when the plants were placed in the growth chamber until the FBV stage. At FBV stage the plants were randomly assigned to one of the two following light treatments. The first treatment, hereafter referred to as “high PPFD,” corresponded to exposure to a continuous PPFD of 529 ± 6 μmol m^-2^ s^-1^ until 14 days after the FBV stage. In the second treatment, hereafter referred to as “low PPFD,” the plants were exposed to 89 ± 1 μmol m^-2^ s^-1^ from the FBV stage to 14 days after the FBV stage. PPFD was measured with a horizontal cosine corrector quantum sensor (LI-190 Quantum Sensor, LI-COR, Lincoln, NE, United States).

### Quantification of Endogenous Sugars and Phytohormones

The fourth internode from the apex (not counting the peduncle, see Supplementary Material 2A) was collected at the FBV stage (day 0), days 4 and 8 after the FBV stage on plants grown under high or low PPFD. Samples were collected 6 h after the beginning of the light period, immediately frozen in liquid nitrogen and stored at -80°C. Then they were lyophilized and crushed. Sucrose, glucose, fructose, and starch (enzymatic digestion step) were determined by colorimetry (Konelab), as presented in Supplementary Material 3. CKs, free ABA, and free IAA concentrations were determined as in Li-Marchetti et al. (2015). Sugar and hormone contents were determined from four replicates with five plants per light treatment each.

### Exogenous Supply of Chemicals

#### Methods for Enriching Plant Tissues

For all chemicals and concentrations, except sugars at high concentrations, the cotton-wick method was used to supply the compounds directly into the stem, as described in Mahieu et al. (2009). The compounds were stored in the form of a liquid solution in a reservoir and brought to the plant by a cotton wick that went through the stem through an 0.5-mm hole positioned 5 mm below the fourth node (all the organs along the primary shoot are counted downward from the apex) (Supplementary Materials 2B,C). The wick was protected from desiccation and the reservoir was re-filled with the solution if needed.

At concentrations equal or above 300 mM, the sugars crystallized along the cotton wick, so they were supplied through the leaf rachis (Lin et al., 2011). The leaflets of the fourth leaf from the top were removed, except for the two proximal leaflets, and the rachis was rapidly immersed in a liquid solution in a 1.5-ml reservoir. After 1 week the rachis was cut 0.5 cm lower. The reservoirs were re-filled if necessary. Both methods supplied the exogenous compounds continuously.

#### Sugar, CK, and ABA Supply

All chemicals were supplied from the FBV stage onward. Sugars and CKs were provided to the plants grown under the low PPFD light treatment to test if they could relieve the bud outgrowth inhibition induced by low PPFD. Sugars were provided mainly as sucrose, which is the circulating form, but also as glucose because these two forms can trigger bud outgrowth in isolated rose nodes grown *in vitro* (Rabot et al., 2012). Under sucrose 50, 100, 200 mM and glucose 100 mM, mannitol 100 mM was used as an osmotic control; under sucrose 300, 600, 800 mM and glucose 600 mM, mannitol 300 mM served as a control. Mannitol is not metabolized by *R. hybrida* and does not stimulate bud outgrowth (Henry et al., 2011; Rabot et al., 2012). These concentrations are typical of phloem sap (Nadwodnik and Lohaus, 2008; Merchant et al., 2009) and are either equal to or higher than those previously shown to be able to trigger bud outgrowth in isolated rose internodes (Henry et al., 2011; Rabot et al., 2012; Barbier et al., 2015).

Synthetic CKs were supplied as 6-benzylaminopurine (BAP) at 50 or 500 μM, solubilized in water with NaOH 40 mM. The control consisted of NaOH 40 mM alone. The concentrations of 50 and 500 μM BAP were within the range of synthetic CKs used in pea and different woody species (Dun et al., 2012; Ni et al., 2015).

To test whether ABA could inhibit bud outgrowth in favorable conditions, ABA was supplied at 1.25 and 2.5 mM with NaOH 40 mM to plants grown continuously under a high PPFD of 350 μmol m^-2^ s^-1^ provided by white LED tubes. The control was an ABA-free solution of NaOH 40 mM. To test whether ABA could also inhibit bud outgrowth under low PPFD, ABA was supplied under low PPFD together with CKs, because buds grew out in plants supplied with CKs under the low PPFD (see “Results” section). ABA and CKs were supplied in the form of solutions containing BAP 500 μM + ABA 1.25, 2.5 or 5.0 mM + NaOH 40 mM. The control contained BAP 500 μM + NaOH 40 mM. High concentrations of ABA were applied as endogenous ABA contents were much higher than endogenous CK contents (see “Results” section).

### ^13^C-Labeled Sugar Uptake Studies

To check that sugars supplied by both the cotton-wick method and the rachis-feeding method were indeed delivered to the stem tissues, the plants grown under low PPFD were supplied with labeled sucrose. Labeled ^13^C-sucrose (ID 605417_Aldrich, Sigma–Aldrich, Darmstadt, Germany) was incorporated at 3% in a solution of 200 mM sucrose supplied by a cotton wick just below node 4, or incorporated at 3.4% in a solution of 600 mM sucrose supplied through the rachis of leaf 4. Water supplied either by a cotton wick or through the rachis served as a control. Exogenous supply started at the FBV stage onward. Days 4 and 8 after the FBV stage, the third, fourth, and fifth internodes from the apex (not counting the peduncle, Supplementary Material 2A) were sampled as described previously. Total carbon and the ^13^C:^12^C ratio were determined for each sample using an elemental analyzer (NA 1500 NCS; Carlo Erba, Milan, Italy) coupled with a Delta-S isotope ratio mass spectrometer (Finnigan-Mat; Thermoquest Corp., San Jose, CA, United States). δ^13^C was calculated as:

$$\delta^{13}C(‰)=(\text{R sample/R reference-1})\times 1000$$

where R is the ratio of heavy-to-light isotope, and the R reference for ^13^C in the PDB standard is 0.0112372.

### Growth Measurements of the Different Buds along the Primary Axis

Three to six times a week from FBV onward, the state (dormant or growing out) of each bud along the primary axis was noted, and bud length was measured with a ruler. Buds were considered to have grown out when at least one visible leaf protruded between the two bud scales (Girault et al., 2008; Roman et al., 2016). The mean bud lengths were calculated from the sizes of outgrowing and dormant buds from a same batch. Fourteen days after the FBV stage, buds were dissected using a stereomicroscope. Bud meristem organogenic activity, which results in the formation of new organs in the bud, was assessed by counting the number of leaves in the bud, leaves being here defined as young leaves plus foliar primordia, excluding the two bud scales. Measurements were performed on 8–16 plants per experimental modality (light treatment and/or chemical exogenous supply), with three biological replicates, except otherwise stated.

### Photosynthesis Measurements

The CO_2_ net assimilation rate was measured as the quantity of CO_2_ assimilated per leaf surface unit per second, and represents the difference between photosynthetic assimilation and respiration. To assess the effect of the light treatments, CO_2_ net assimilation rates were measured both in a leaf still rapidly expanding and partially unfolded at the FBV stage (leaf 4 from the apex), and in a mature fully unfolded leaf (leaf 6 from the apex), on five plants, 3 h before and 3 h after the switch in light intensity, and then once a day for 14 days, 6 h after the beginning of the light period.

To test if the CK supply or the CK – ABA supply affected photosynthesis in plants grown under low PPFD, CO_2_ net assimilation rates were measured in leaves of plants supplied according to the following conditions: CK 500 μM + NaOH 40 mM, CK 500 μM + ABA 1.25, 2.5, 5.0 mM + NaOH 40 mM, control NaOH 40 mM. CO_2_ net assimilation rates were measured in the leaves positioned immediately above and below the supply point (leaves 4 and 5, respectively, Supplementary Materials 2A,B) on four plants per condition, every 2 days from FBV onward, 6 h after the beginning of the light period. Leaf positions were chosen so as to maximize the likelihood to observe an effect of the exogenous supply if it existed.

All measurements of CO_2_ net assimilation rates were performed using an infrared gas analyser, Li-Cor-6400 (Li-Cor Inc., Lincoln, NE, United States), at a temperature of 18°C, a relative humidity of 64%, a cuvette air flow rate of 300 ml min^-1^, and an ambient CO_2_ concentration of 400 μmol mol^-1^. PPFDs of 540 and 90 μmol m^-2^ s^-1^ were provided to the measured leaves for the plants grown under high and low PPFD, respectively, by a mixture of red and blue LEDs.

### Statistical Analysis

Statistical analyses were performed using R software for Windows (R Core Team, 2014). Groups were compared using one-way ANOVA (α = 0.05) followed by Tukey’s test when the ANOVA indicated significant differences at *P* < 0.05 and there were more than two groups.

## Results

### Decreasing PPFD Inhibits Axillary Bud Outgrowth All along the Stem

**FIGURE 1:**
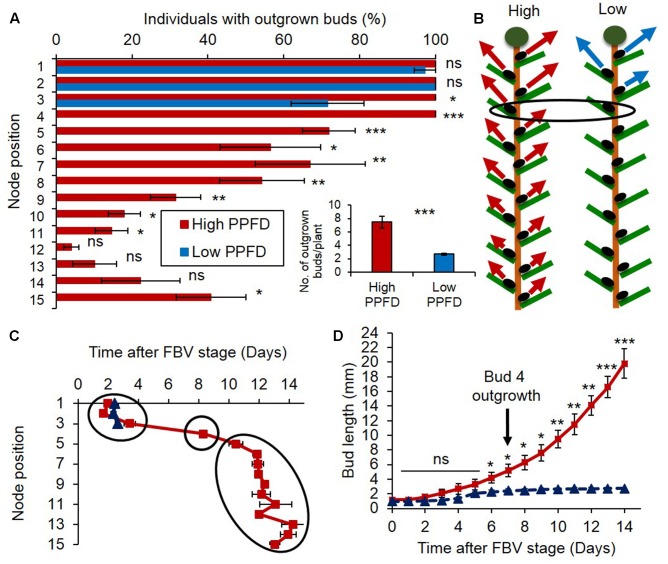
Impact of PPFD on bud outgrowth pattern along rose primary axis. **(A)** Percentage of individuals with outgrown buds 14 days after FBV (floral bud visible) stage according to node position along the shoot. **(A-inset)** Total number of buds per plant that grew out 14 days after FBV. **(B)** Schematic representation of the bud outgrowth pattern of plants grown under high or low PPFD treatments: at each node position an arrow indicates that the bud grows out whereas an absence of arrow indicates bud inhibition, the longer the arrow, the higher the percentage of individuals with outgrown buds at this node position. **(C)** Date of bud outgrowth according to bud location. **(D)** Elongation of bud 4 along time under high or low PPFD treatment. On day 8, first leaf protrudes out bud scales, marking bud outgrowth. Buds are numbered basipetally along the stem. Each point represents the average of three repetitions, with 10 plants per repetition. Error bars represent the standard error of the mean (SE). ANOVA: ^∗^*P* < 0.05; ^∗∗^*P* < 0.01; ^∗∗∗^*P* < 0.001.

The number of outgrown buds per plant was much lower under the low PPFD treatment than under the high PPFD treatment (**Figure 1A-inset** and Supplementary Figure S1). Under high PPFD buds grew out all along the stem, displaying an acrotonic pattern with a decreasing outgrowth rate from the apical to the basal part of the stem (**Figures 1A,B**). The time course of bud outgrowth was sequential, starting from the two uppermost apical buds 2 days after the FBV stage and proceeding downward (**Figure 1C**). The uppermost two apical buds did not respond to a reduction of PPFD because they are sylleptic buds whose outgrowth is independent of environmental conditions (Huché-Thélier et al., 2011; Demotes-Mainard et al., 2013b). But, the outgrowth rate of the third topmost bud was reduced as compared to high PPFD conditions (72% vs. 100%), and outgrowth of all the buds from the fourth topmost bud and below was totally inhibited (**Figures 1A,B**). Bud 4 displayed the most contrasted outgrowth behavior between PPFD treatments. While under high PPFD all buds 4 had grown out 8 days after the FBV stage on average, none grew out in low PPFD (**Figures 1A,C**). Elongation of bud 4 was rapid and continuous under high PPFD, whereas it was slow and stopped rapidly under low PPFD (**Figure 1D**).

### Decreasing PPFD Reduces Sugar Contents in the Internode, but Local Sugar Supply Is Not Sufficient to Restore Bud Outgrowth

**FIGURE 2:**
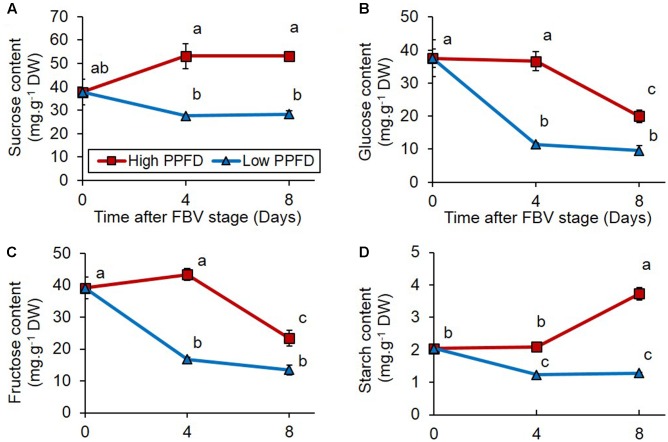
Impacts of PPFD on sugar content of internode 4. **(A)** Sucrose, **(B)** glucose, **(C)** fructose, and **(D)** starch contents of internode 4 at FBV stage, 4 and 8 days after FBV stage. Internode 4, that supports bud 4, is the fourth internode counted from the apex, excluding the flower peduncle. Each data represents the average of four repetitions. Error bars represent standard error of the mean (SE). Different letters indicate significant differences between treatments and times (ANOVA followed by a Tukey test, *P* < 0.05).

To determine if sugars are involved in mediating the effect of PPFD on bud outgrowth, we analyzed the sugar content of internode 4, which is adjacent to the bud displaying the most contrasted response to PPFD treatments (**Figures 1A,B**). Internode 4 displayed a lower content in sucrose, glucose, fructose, and starch under low PPFD than under high PPFD days 4 and 8 after the FBV stage (**Figure 2**). Day 8 was the time when bud 4 grew out under high PPFD (**Figure 1C**), and day 4 was the time when we presume that conditions may have affected the inducement of bud 4 outgrowth since 4 days are required for bud outgrowth after apical dominance has been relieved under favorable light conditions in this cultivar (Girault et al., 2010). The correlation between sugar content in internode 4 and bud 4 outgrowth thus suggests that a low sugar level in the node may be involved in bud outgrowth inhibition under low PPFD.

**FIGURE 3:**
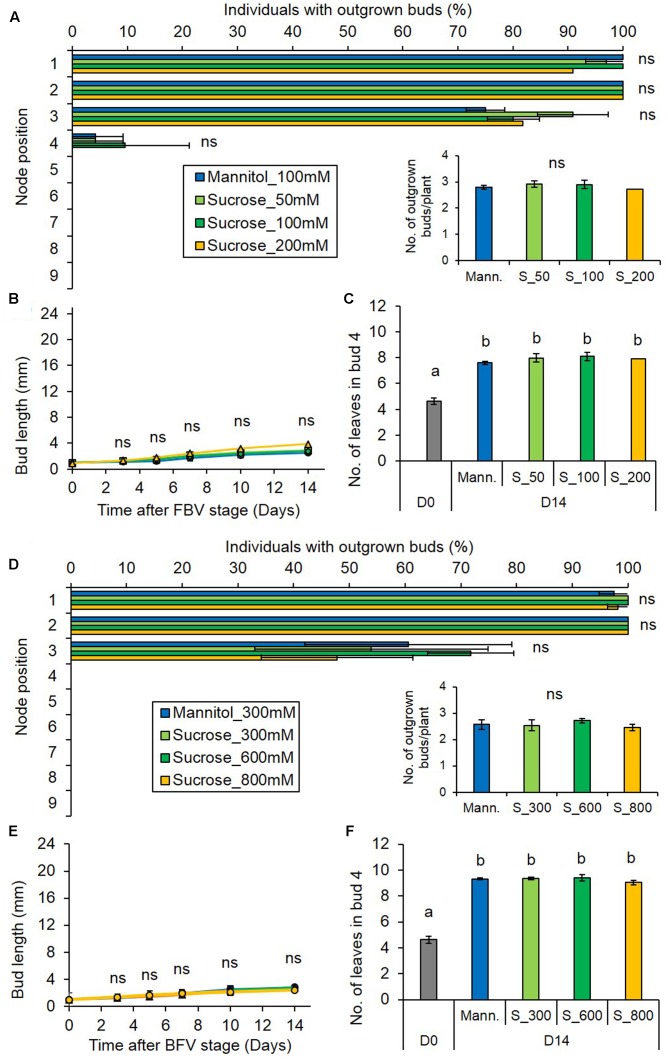
Impact of sucrose supply on bud outgrowth pattern of plants grown under the low PPFD treatment. Sucrose was supplied through the stem below node 4 at 50, 100, and 200 mM **(A–C)** or through the rachis of leaf 4 at 300, 600, and 800 mM **(D–F)** from FBV stage onward, and mannitol (100 mM **A–C** or 300 mM **D–F**) was used as non-metabolizable control. **(A,D)** Effect of sucrose supply on the percentage of individuals with outgrown buds 14 days after FBV according to node position along the shoot. **(A-inset,D-inset)** Total number of buds per plant that grew out 14 days after FBV. **(B,E)** Elongation of bud 4 along time. **(C,F)** Number of leaves in bud 4 at FBV stage (day 0) and 14 days after FBV (day 14). Buds are numbered basipetally along the stem. Each point represents the average of three repetitions. Error bars represent the standard error of the mean (SE). Different letters indicate significant differences between sugar supplies and times, ns indicates an absence of significant difference (ANOVA, followed by a Tukey test for **C**, **F**, *P* < 0.05).

To investigate this hypothesis, we tested if local sugar supply could relieve bud inhibition under reduced PPFD. Sugars were supplied to plants either through a cotton wick (50, 100, and 200 mM) or through the rachis for the higher concentrations (300, 600, and 800 mM). Using labeled ^13^C-sucrose, we demonstrated that sugars were indeed delivered into the stem tissues by these two methods (Supplementary Figures S2A,B). Sucrose supplied under low PPFD did not promote bud outgrowth, nor did it change the gradient of bud outgrowth along the stem as compared to the control, whatever the concentration (**Figures 3A,A-inset,D,D-inset**). Neither did it stimulate bud elongation (**Figures 3B,E**) or leaf formation into the bud (**Figures 3C,F**). Similar results were observed when glucose (100 or 800 mM) instead of sucrose was supplied (Supplementary Figures S3A–F). Altogether, these results show that although low PPFD leads to reduced sugar levels in the internode, the bud outgrowth inhibition induced by low PPFD cannot be solely relieved by local sugar supply. We therefore investigated whether CKs and ABA may limit bud outgrowth under low PPFD.

### Decreasing PPFD Reduces CK Contents in the Internode, and CK Supply Can Relieve Low PPFD-Induced Bud Inhibition

**FIGURE 4:**
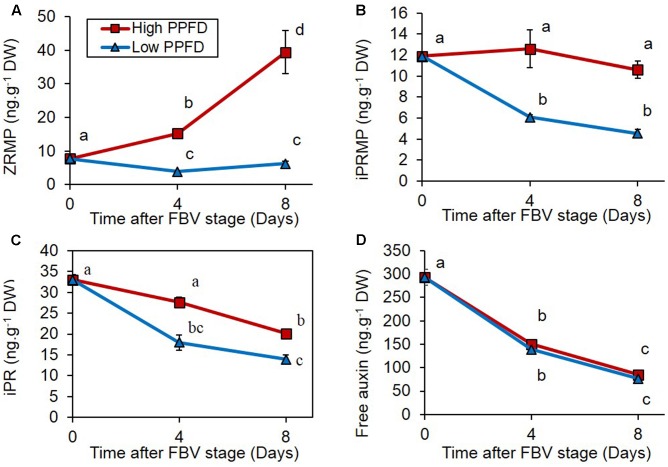
Effects of PPFD on CK contents of internode 4. **(A)** Zeatin riboside-5′-monophosphate (ZRMP), **(B)** isopentenyl adenosine-5′-monophosphate (iPRMP), **(C)** isopentenyl adenine riboside (iPR), **(D)** free auxin (IAA) contents of internode 4 at FBV stage, 4 and 8 days after FBV stage. Internode 4, that supports bud 4, is the fourth internode counted from the apex, excluding the flower peduncle. Each data represents the average of four repetitions. Error bars represent standard error of the mean (SE). Different letters indicate significant differences between treatments and times (ANOVA followed by a Tukey test, *P* < 0.05).

Cytokinin (zeatin riboside-5′-monophosphate, ZRMP; isopentenyl adenosine-5′-monophosphate, iPRMP; isopentenyl adenine riboside, iPR) contents were measured in internode 4 under high and low PPFD treatments 4 and 8 days after the FBV stage. ZRMP and iPRMP are intermediate forms in the CK biosynthesis pathway, and iPR is an active form. The three CK forms followed different temporal dynamics under high PPFD: the content in ZRMP increased over the 8 days following the FBV stage, whereas the content in iPRMP was stable, and the content in iPR decreased over time (**Figures 4A–C**). Low PPFD reduced the content in all three CK forms in internode 4, 4 and 8 days after the FBV stage (**Figures 4A–C**), whereas the free IAA content in internode 4 was not affected by the PPFD level (**Figure 4D**).

**FIGURE 5:**
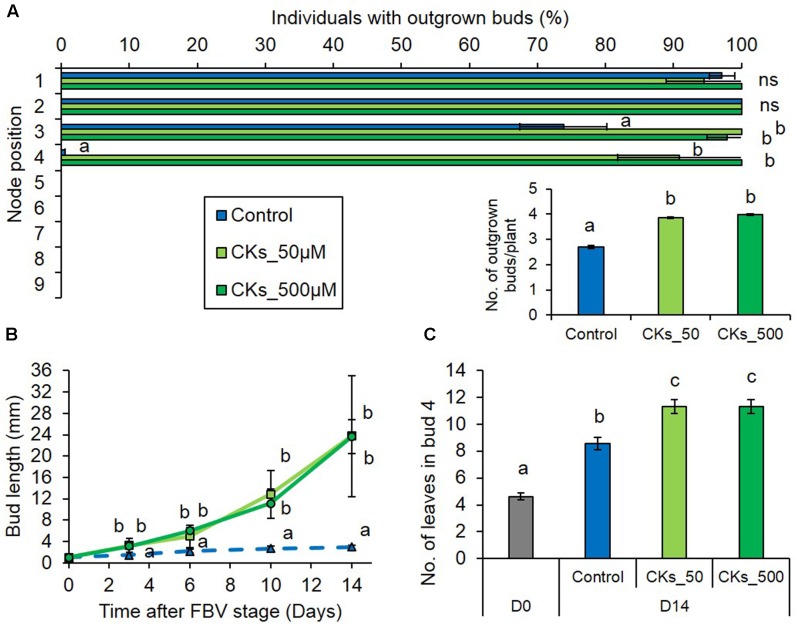
Effects of synthetic CK (BAP) supply on bud outgrowth pattern of plants grown under the low PPFD treatment. CKs were supplied through the stem below node 4 from FBV stage onward as solutions with 50 or 500 μM BAP, a solution without CK was used for the control. **(A)** Effect of CK supply on the percentage of individuals with outgrown buds 14 days after FBV according to node position along the shoot. **(A-inset)** Total number of buds per plant that grew out 14 days after FBV. **(B)** Elongation of bud 4 along time. **(C)** Number of leaves in bud 4 at FBV stage (day 0) and 14 days after FBV (day 14). Buds are numbered basipetally along the stem. Each point represents the average of three repetitions. Error bars represent the standard error of the mean (SE). Different letters indicate significant differences between CK supplies and times, ns indicates an absence of significant differences (ANOVA followed by a Tukey test, *P* < 0.05).

In order to assess if CKs may be players in the regulation of the bud outgrowth pattern by PPFD, we tested if exogenous CKs could relieve the bud outgrowth inhibition induced by low PPFD. Synthetic CK BAP was supplied to internode 4 of plants grown under low PPFD using cotton wicks. When 50 and 500 μM CK were supplied, the number of buds that grew out per plant increased significantly as compared to the control (**Figure 5A-inset**). Increased outgrowth was observed locally close to the supply point for bud 4, but also at a distance for bud 3 (**Figure 5A**). Bud 4, whose outgrowth was totally inhibited under low PPFD in the absence of CK, displayed rates of 91 and 100% outgrowth when the plants were supplied with 50 and 500 μM of CKs, respectively, under low PPFD. Under low PPFD again, outgrowth of bud 4 was observed 7.78 ± 0.44 and 7.49 ± 0.39 days after the FBV stage in plants supplied with CKs at 50 and 500 μM, respectively, and bud 4 elongation was increased by exogenous CKs (**Figure 5B**), giving a similar date of outgrowth and dynamics of elongation as observed under high PPFD (**Figures 1C,D**). CK supply under low PPFD also increased the number of leaves formed into the bud (**Figure 5C**). Taken together, these results suggest that low PPFD inhibition of bud outgrowth is mediated at least partly through a lower stem CK content.

### Low PPFD Increases the ABA Content, and ABA Supply Can Inhibit Bud Outgrowth under High PPFD and Antagonizes CK Stimulation of Bud Outgrowth under Low PPFD

**FIGURE 6:**
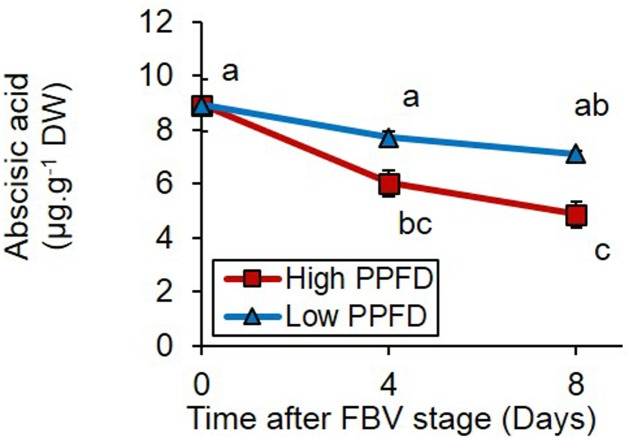
Effects of PPFD treatments on free ABA content of internode 4 at FBV stage, 4 and 8 days after FBV stage. Internode 4, that supports bud 4, is the fourth internode counted from the apex, excluding the flower peduncle. Each data represents the average of four repetitions. Error bars represent standard error of the mean (SE). Different letters indicate significant differences between treatments and times (ANOVA followed by a Tukey test, *P* < 0.05).

Measurements of free ABA contents in the adjacent internode to bud 4 revealed that inhibition of bud outgrowth by low PPFD was also correlated to the maintenance of higher levels of ABA 4 and 8 days after the FBV stage (**Figure 6**).

**FIGURE 7:**
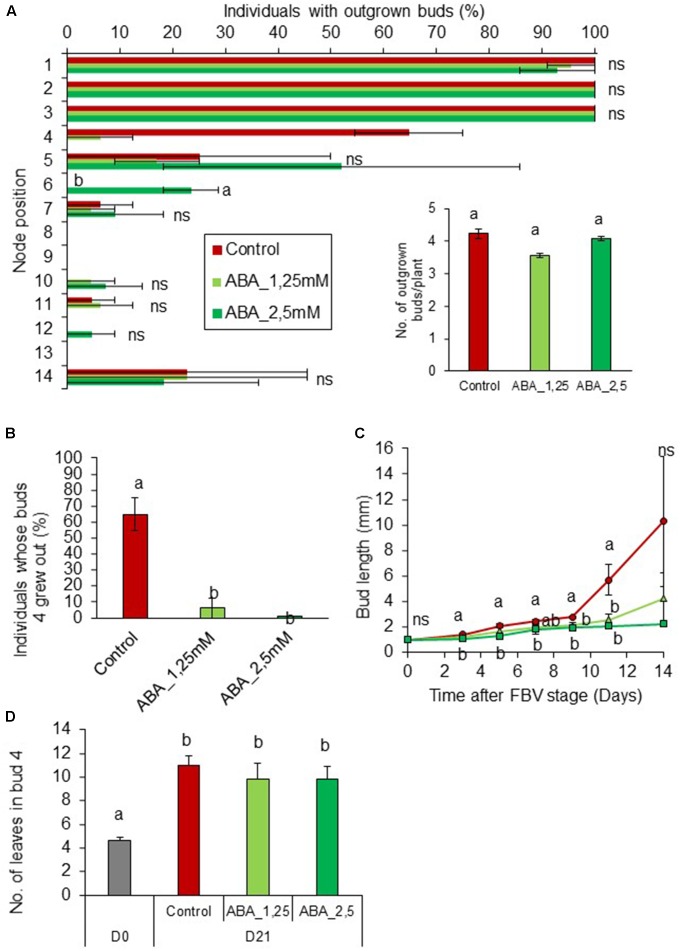
Impact of ABA supply on bud outgrowth pattern of plants grown under high PPFD. ABA (1.25 and 2.5 mM) was supplied by a cotton wick through the stem below node 4 from FBV stage onward, a solution without ABA was used for the control. **(A)** Effect of ABA supply on the percentage of individuals with outgrown buds 14 days after FBV according to node position along the shoot. **(A-inset)** Total number of buds per plant that grew out 14 days after FBV. **(B)** Percentage of individuals whose buds 4 grew out 14 days after FBV stage. **(C)** Elongation of bud 4 along time. **(D)** Number of leaves in bud 4 at FBV stage (day 0) and 21 days after FBV (day 21). Buds are numbered basipetally along the stem. Each point represents the average of two repetitions. Error bars represent the standard error of the mean (SE). Different letters indicate significant differences between ABA supplies, ns indicates an absence of significant difference (ANOVA followed by a Tukey test, *P* < 0.05).

To assess whether ABA could be involved in mediating PPFD control of bud outgrowth, we tested whether exogenous supply of ABA (0, 1.25, and 2.5 mM) to internode 4 could inhibit bud outgrowth under high PPFD (350 μmol m^-2^ s^-1^). ABA supply at both 1.25 and 2.5 mM had a strong inhibitory effect on the outgrowth and elongation of bud 4 (**Figures 7B,C**) but not on the number of leaves formed per bud (**Figure 7D**). Interestingly, outgrowth of buds located below the point of ABA supply increased (**Figure 7A**), so that the total number of outgrown buds per plant was not affected (**Figure 7A-inset**).

**FIGURE 8:**
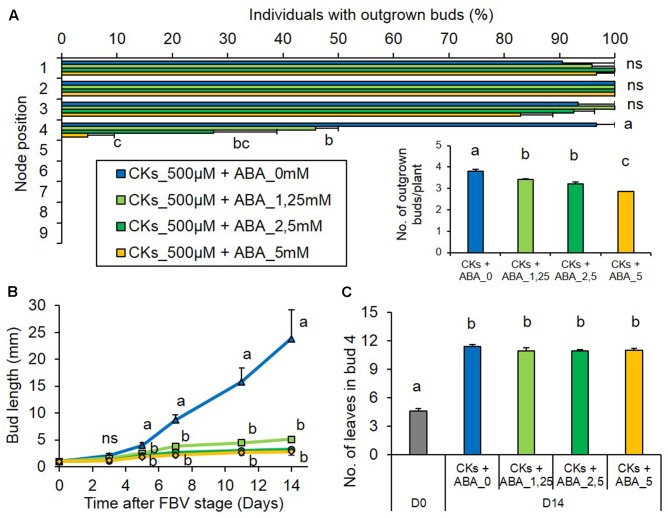
Effects of ABA supplied together with CKs on bud outgrowth pattern of plants grown under the low PPFD treatment. ABA (0, 1.25, 2.5, and 5 mM) and CKs (BAP 500 μM) were supplied together through the stem below node 4 from FBV stage onward, a solution with CKs but without ABA was used for the control. **(A)** Effect of ABA supply on the percentage of individuals with outgrown buds 14 days after FBV according to node position along the shoot. **(A-inset)** Total number of buds per plant that grew out 14 days after FBV. **(B)** Elongation of bud 4 along time. **(C)** Number of leaves in bud 4 at FBV stage (day 0) and 14 days after FBV (day 14). Buds are numbered basipetally along the stem. Each point represents the average of three repetitions. Error bars represent the standard error of the mean (SE). Different letters indicate significant differences between ABA supplies and times, ns indicates an absence of significant difference (ANOVA, followed by a Tukey test, *P* < 0.05).

To test whether a high ABA stem content could inhibit bud outgrowth under low PPFD, ABA was supplied under low PPFD together with CK, a promoter of outgrowth. ABA antagonized the stimulating effect of CK in a dose-dependent manner and decreased bud 4 outgrowth (**Figures 8A,A-inset**). With the lowest ABA concentration, only 46% of these buds grew out and their outgrowth was delayed by 2 days, occurring only 10.2 ± 0.4 days after FBV, whereas with the highest ABA concentration only 4.7% of buds 4 grew out. In the presence of CKs, ABA also drastically limited the elongation of bud 4 (**Figure 8B**). With the highest ABA concentration supplied together with CK, the frequencies of bud 4 outgrowth and the kinetics of bud 4 elongation were very similar to those observed under low PPFD without any exogenous supply (**Figures 1**, **8**). ABA had no effect on the number of leaves formed into the bud (**Figure 8C**). The buds below the supply point did not grow out, whatever the ABA concentrations (**Figure 8A**).

Altogether, these results indicate that low PPFD maintains high ABA levels in the stem and that stem ABA can inhibit bud outgrowth under low and high PPFD in rose. They suggest that the high ABA stem contents induced by low PPFD may contribute to the inhibition of bud outgrowth, notably by antagonizing the promoting effect of CKs on bud outgrowth.

### Neither CK Stimulation of Bud Outgrowth under Low PPFD Nor ABA Antagonism of CK Stimulation under Low PPFD Require a Change in CO_2_ Net Assimilation

**FIGURE 9:**
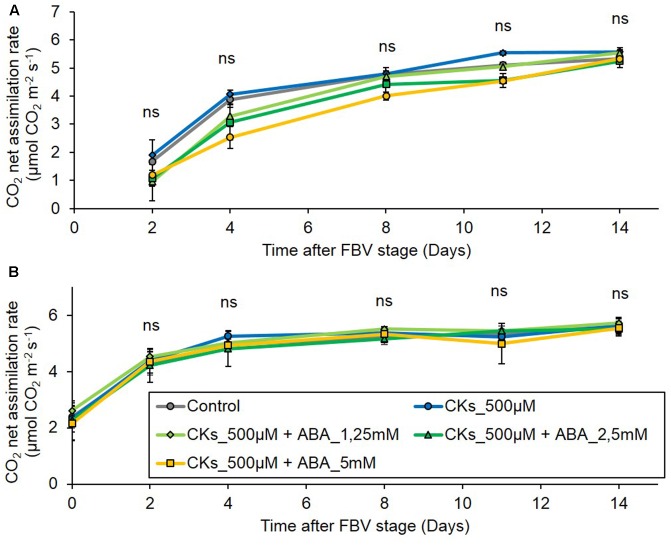
Effects of CK supply or of ABA plus CK supply to plants grown under the low PPFD treatment on leaf CO_2_ net assimilation rate. CKs (BAP 500 μM) were supplied alone or together with ABA (1.25, 2.5, and 5 mM) through the stem below node 4 from FBV stage onward, a solution without CKs nor ABA was used for the control. Leaves were exposed to 90 μmol m^-2^ s^-1^ PPFD, CO_2_ net assimilation rate was measured on **(A)** leaf 4, located above the supply point and **(B)** leaf 5, located below the supply point. Leaves are numbered basipetally along the shoot. Each point represents the average of four plants. Error bars represent standard error of the mean (SE). There is no significant difference between treatments within a leaf and a time point (ANOVA, *P* > 0.05).

The decrease in PPFD from the FBV stage onward markedly and durably reduced photosynthetic CO_2_ net assimilation rates as compared to high PPFD, both in mature and in still rapidly expanding leaves (Supplementary Figure S4). The literature indicates that ABA may dampen photosynthesis (Raschke and Hedrich, 1985; Zhou et al., 2006), and that CKs alone or CKs and ABA together may affect photosynthesis in a way that varies with the species, the conditions of application and the plant growth conditions (Pospíšilová, 2003; Pospíšilová and Bat’ková, 2004; Shu-Qing et al., 2004). We studied whether the effect of CKs and ABA on bud outgrowth passed through an indirect effect on photosynthetic assimilation in our conditions. Over the 2 weeks following the FBV stage, CO_2_ net assimilation rates of leaves 4 and 5 of plants grown under low PPFD were affected neither by CK supply alone nor by combined CK and ABA supply, whatever the ABA concentration (**Figures 9A,B**). These results indicate that the effects of CKs and ABA on the bud outgrowth pattern under low PPFD are not directly mediated by changes in photosynthetic assimilation.

## Discussion

### The Responses of Bud Outgrowth to PPFD Are Preceded by Changes in CK, ABA, and Sugar Stem Contents

The reduced PPFD applied from the FBV stage onward reduced the number of outgrowing buds, consistently with previous results in rose (Bredmose, 1995; Furet et al., 2014; Wubs et al., 2014). To address the question of how the CK, ABA, and sugar regulation pathways take part in the control of the branching pattern by light intensity, we focused on bud 4 that displayed the most contrasted fate with 100% or 0% outgrowth under high or low PPFD, respectively. Changes in PPFD led to variations in endogenous CK, ABA, and sugar contents in internode 4, consistent with bud outgrowth response to PPFD: CKs and sugars, which promote bud outgrowth, had their levels decreased under low PPFD, while ABA, an outgrowth inhibitor, had its content increased. The effect of PPFD on the CK stem content was consistent with the effect observed following exposure to light or darkness by Roman et al. (2016), but information on the effects of PPFD on the ABA stem content was lacking. The reduction in sugar stem content was consistent with the severe decrease in photosynthesis observed under low PPFD but may also have involved changes in partitioning. In addition, the variations in endogenous CK, ABA, and sugar contents in internode 4 preceded the outgrowth of the adjacent bud under high PPFD by at least 4 days; this supports the hypothesis that these chemicals could indeed contribute to the regulation of bud outgrowth. In rose, modifications in sugars and hormone (CK) levels and in expression of genes involved in bud outgrowth regulation were reported to be triggered before the occurrence of bud outgrowth (Girault et al., 2010; Henry et al., 2011; Rabot et al., 2012; Barbier et al., 2015; Roman et al., 2016).

### The CK Stem Content Appears to Be a Key Factor Mediating PPFD Regulation of Bud Outgrowth Pattern along the Stem in an Intact Plant

Despite the major role of CKs in promoting bud outgrowth (Dun et al., 2012; Müller et al., 2015), studies investigating their involvement in the regulation of bud outgrowth by light are scarce. Stem endogenous CKs dropped under low PPFD concomitantly to bud inhibition and conversely CK supply to plants grown under low PPFD relieved the inhibition of bud outgrowth. CK supply stimulated bud meristem organogenic activity and bud elongation, that are the two fundamental processes of bud outgrowth. This stimulation happened in such a way that CK supply under low PPFD resulted in the same timings of bud 4 outgrowth and the same kinetics of bud 4 elongation as those observed under high PPFD. Altogether, these results are consistent with our previous findings showing that CKs are initial targets of light in the regulation of bud outgrowth in decapitated defoliated plants (Roman et al., 2016). They further bring evidence that (i) CKs allow buds to be relieved from ecodormancy due to unfavorable environmental conditions (low PPFD) and from paradormancy (apical dominance and other correlative inhibitions, Zieslin and Halevy, 1976; Domagalska and Leyser, 2011); (ii) a reduction of CK levels not only mediates repression of bud outgrowth induced by total darkness but also by low PPFD, as can be encountered under normal daylight conditions.

The stimulating effect of CKs on bud meristem organogenic activity under low PPFD is consistent with the ability of CKs to rescue organogenic activity under darkness in shoot apical meristems of tomato and rose (Yoshida et al., 2011; Roman et al., 2016).

### The Changes in Stem CK Contents in Response to PPFD Are Not Mediated by Changes in IAA Contents

Knowing that stem IAA can downregulate stem CK contents (Tanaka et al., 2006; Shimizu-Sato et al., 2009), we wondered if the changes in stem CKs in response to PPFD may have resulted, at least partly, from a change in stem IAA. Knowledge on the effects of PPFD on stem auxin contents is poor, and the results obtained in various species and growth conditions are conflicting (Kurepin et al., 2006, 2007, 2008; Hersch et al., 2014). Here, the absence of difference in IAA contents in internode 4 between the two PPFD treatments indicates that the changes in stem CK content was not a consequence of changes in stem IAA levels. Roman et al. (2016) also observed changes in CK stem contents in response to light in the absence of variations in IAA content, but the stem IAA levels in their plant system were extremely low due to decapitation.

Both stem-synthesized CKs and root-derived CKs can contribute to bud outgrowth regulation, with conflicting results between species (Faiss et al., 1997; Tanaka et al., 2006; Müller et al., 2015). In our study we can assume that the regulation of the CK stem content by PPFD may result at least partly from local regulation of the CK metabolism in the stem, since CK synthesis genes are downregulated and CK degradation genes are upregulated in the stem of plants grown in darkness as compared to light (Roman et al., 2016). Regulation of the CK stem content may also partly result from changes in xylem-carried CKs coming from the roots, since in tobacco plants shade reduces xylem carried CKs by reducing the transpiration rate (Boonman et al., 2007).

### The High ABA Content in the Stem Induced by Reduced PPFD Can Inhibit Bud Outgrowth along the Stem and Antagonize CKs

Our results suggest that the high stem ABA content induced by low PPFD contributed to the regulation of bud outgrowth. Exogenous ABA supply to the stem inhibited bud outgrowth in rose under high PPFD, i.e., in environmental conditions favorable to outgrowth, in accordance with previous data in Arabidopsis, pea, *Ipomoea nil*, and tomato (Arney and Mitchell, 1969; Chatfield et al., 2000; Cline and Oh, 2006; Yao and Finlayson, 2015). To investigate further the role of ABA in mediating bud outgrowth regulation by PPFD, we showed that ABA could also inhibit bud outgrowth under low PPFD. ABA was able to antagonize the promotive effect of exogenous CKs on bud outgrowth in a dose-dependent manner under low PPFD. The antagonistic role of ABA and CKs had been evidenced in other processes such as stomatal regulation, seed germination or seedling development (Dodd, 2005; Wang et al., 2011; Guan et al., 2014), but such a dose-dependent antagonism was evidenced in this study for the first time in the regulation of bud outgrowth. Altogether, our results suggest that the effect of PPFD on bud outgrowth may be complex and that reduced PPFD inhibits bud outgrowth both by reducing the stem content in CKs (a branching inducer) and by maintaining a high content in stem ABA (a branching repressor). The high ABA content can dampen the effects of the remaining CKs. ABA was already known to mediate the response to the R:FR ratio or the photoperiod in Arabidopsis (Reddy et al., 2013; Yao and Finlayson, 2015; González-Grandío et al., 2017; Holalu and Finlayson, 2017). Our results extend its role to the mediation of environmental regulation of bud outgrowth by another light signal and species. In addition, they also point out the contribution of stem ABA in controlling bud outgrowth, and complement the previous works that focused on bud ABA.

Interestingly, ABA inhibited bud outgrowth and impaired bud elongation, but did not affect meristem organogenic activity in the bud, irrespective of the PPFD level. In contrast, CKs stimulated bud meristem organogenic activity, which indicates that the antagonistic effect of ABA and CKs was limited to certain bud outgrowth-related processes.

### Supplying Sugars Close to the Bud Cannot Alone Relieve Low PPFD-Induced Bud Inhibition

Under low PPFD, exogenous supply of both sucrose and glucose close to the bud failed to promote bud outgrowth in intact plants. These results suggest that sugars are not the main limiting factor for bud outgrowth in intact plants under low PPFD, but would act downward other factors, at least CKs and ABA. These results are consistent with those obtained in the upper bud of decapitated defoliated plants grown under darkness (Roman et al., 2016).

Cytokinins have positive effects on source-sink organ activities (Cowan et al., 2005; Peleg et al., 2011). In our case the CK supply that promoted outgrowth under low PPFD is unlikely to have acted on the global sugar content per plant as it did not change photosynthetic assimilation. CK supply to the stem is known to increase the sugar sink strength of the bud (Roman et al., 2016), in agreement with CK-mediated upregulation of sugar sink strength in other tissues (Roitsch and González, 2004). Thus, we can hypothesize that one action of CK supply in the vicinity of the bud under low PPFD was to increase bud sugar sink strength and allow buds to better compete for sugars at the expense of other plant organs. Unlike CKs, ABA affects negatively sink strength by limiting the availability of nutrients and energy (Garciarrubio et al., 1997; Parish et al., 2012). It would be of great interest to investigate in future works if ABA negatively affects the sink strength of buds and how it interacts with CKs in response to PPFD. Mason et al. (2014) demonstrated that sugar supply alone can promote bud outgrowth when plants are grown in a non-limiting light environment in which the shoot tip’s high demand for sugars is the limiting factor for bud access to sugars. In our experiments the apical organs, that were in rapid expansion (Demotes-Mainard et al., 2013a), most probably intensively competed with buds for sugars too. However, sugar supply to the stem could not trigger outgrowth under low PPFD because PPFD controlled bud outgrowth through an upstream mechanism, involving CK and presumably ABA regulation. We hypothesize that at least part of this control is due to low stem CK content that restricts sugar import into the bud under low PPFD.

### Action of Exogenous CKs and ABA on Bud Outgrowth at Different Locations along the Stem

Exogenous CK supply had an effect on the directly treated bud (bud 4) and on the first bud located above it (bud 3). The absence of effect on the buds located below the supply point is consistent with results on isolated nodes and decapitated plants (Ohkawa, 1984; Chatfield et al., 2000).

Abscisic acid supply in the stem inhibited only the local bud. We did not expect exogenous ABA supply to inhibit lower buds since ABA inhibits outgrowth in isolated nodes only when supplied from the basal side of the stem (Chatfield et al., 2000). The absence of inhibition of bud 3, located apically, may result from the dose of ABA that reached bud 3 and/or from a lower sensitivity of this bud to ABA due to its physiological state. Bud 3 was indeed much less sensitive to PPFD than the buds below, as shown by the outgrowth patterns under low and high PPFD.

When exogenous ABA totally inhibited bud 4 outgrowth under high PPFD, the outgrowth rate of the buds just below it (buds 5 and 6) increased. This increase in outgrowth may be an indirect effect of bud 4 inhibition under high PPFD: bud 4 inhibition by ABA may have both reduced the correlative inhibition exerted on lower buds (Domagalska and Leyser, 2011) and made sugars more available for these lower buds, thereby facilitating their outgrowth under high PPFD (Mason et al., 2014). In isolated internodes of Arabidopsis, ABA supplied from the apical side of the stem reduced inhibition by IAA (Chatfield et al., 2000). Such an effect cannot be ruled out but was not observed in *Ipomoea nil* or in sunflower (Cline and Oh, 2006).

## Conclusion

**FIGURE 10:**
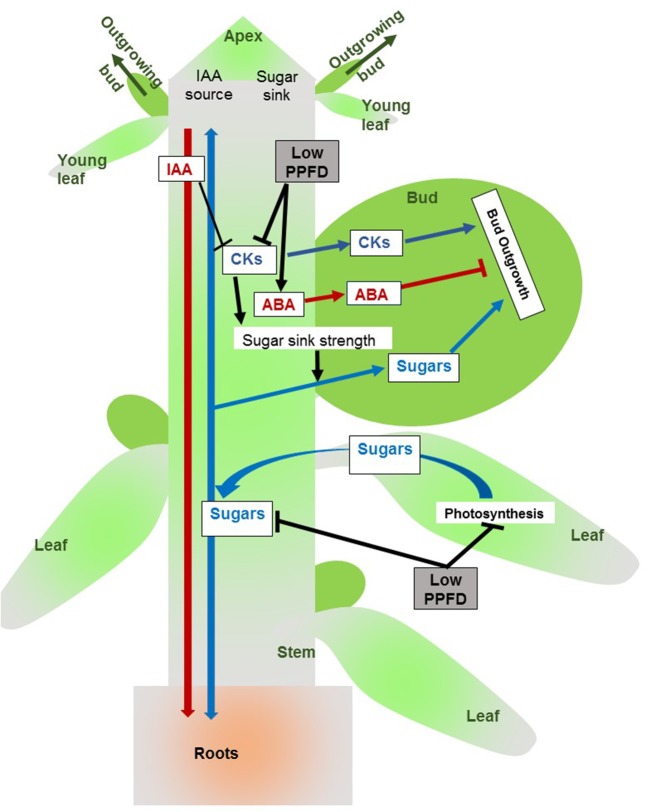
Schematic representation of the relationships between CKs, ABA, IAA, and sugars in the regulation of bud outgrowth by PPFD in an intact rose plant. Low PPFD reduces stem CK content, which appears to be a key limiting factor of bud outgrowth. The level of CKs transported from the stem to the bud, where they stimulate outgrowth, is likely to be dampened under low PPFD. The CK stimulation of bud sugar sink strength, shown in decapitated rose plants (Roman et al., 2016), may also be lowered. Low PPFD maintains a high stem ABA content, ABA presumably enters the bud where it inhibits outgrowth, antagonizing the effects of CKs. Low PPFD also reduces photosynthesis and sugar stem content. However, sugar stem content is not the main limiting factor for bud outgrowth since sugar supply is not able to override bud outgrowth inhibition under low PPFD. The reduced bud sugar sink strength under low PPFD may prevent the use of sugars by the bud for outgrowth. Although IAA is known to dampen stem CK content, low PPFD effect on CK is not mediated through changes in stem IAA content.

This study provides evidence that in intact rose plants reduced PPFD controls bud outgrowth pattern both by reducing the stem CK content and by maintaining a high stem ABA content. These two hormones regulate bud outgrowth antagonistically, but their antagonistic effect is limited to certain bud outgrowth-related processes since bud meristem organogenic activity is solely regulated by CKs. Although the stem sugar content was severely reduced under low PPFD, this drop did not appear to be the triggering factor of bud outgrowth inhibition under low PPFD. A model based on our results and literature is presented in **Figure 10**. Further work will contribute to investigate the relationships between CKs and ABA in the regulation cascade of the bud outgrowth pattern in response to PPFD by investigating at the molecular level regulation pathways between buds showing different sensitivity levels to PPFD variations.

## Author Contributions

AC, NL, SS, SD-M conceived and designed the research. AC, OD, HA, M-DP-G, and SC carried the experimental work, collected and prepared the data. AC conducted statistical analyses. NL, SS, and SD-M directed the work. AC, HR, JB, SS, NL, and SD-M interpreted the data and wrote the paper.

## Conflict of Interest Statement

The authors declare that the research was conducted in the absence of any commercial or financial relationships that could be construed as a potential conflict of interest.

## Abbreviations

ABA: abscisic acid
CKs: cytokinins
FBV: flower bud visible
PPFD: photosynthetic photon flux density
