# Supplementary material for: Cytokinins and Abscisic Acid Act Antagonistically in the Regulation of the Bud Outgrowth Pattern by Light Intensity
Source: Front Plant Sci. 2017 Oct 10;8:1724. doi: 10.3389/fpls.2017.01724 (PMC5641359; doi:10.3389/fpls.2017.01724)
Supplement: Supplementary file 1 [file Presentation_1.pdf]

Supplementary Material

Cytokinins and abscisic acid act antagonistically in the regulation of bud outgrowth pattern by light intensity

Adrien Corot[1], Hanaé Roman[1], Odile Douillet[1], Hervé Autret[1], Maria-Dolores Perez-Garcia[1], Sylvie Citerne[2] Jessica Bertheloot[1], Soulaiman Sakr[1], Nathalie Leduc[1], Sabine Demotes-Mainard[1*]

*** Correspondence:** Dr. Sabine Demotes-Mainard, sabine.demotes-mainard@inra.fr

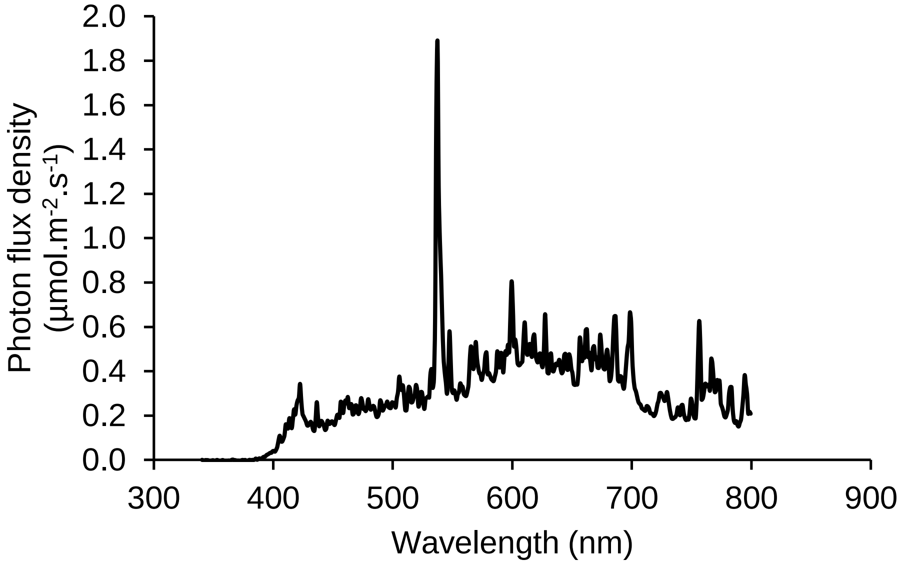

Supplementary material 1. Light spectrum in the growing chambers, as measured with a calibrated spectrophotometer Avaspec-2048-6-RM (Avantes, Apeldoorn, The Netherlands). The R:FR over the ranges (655-665 nm) : (725-735 nm) was 1.99.

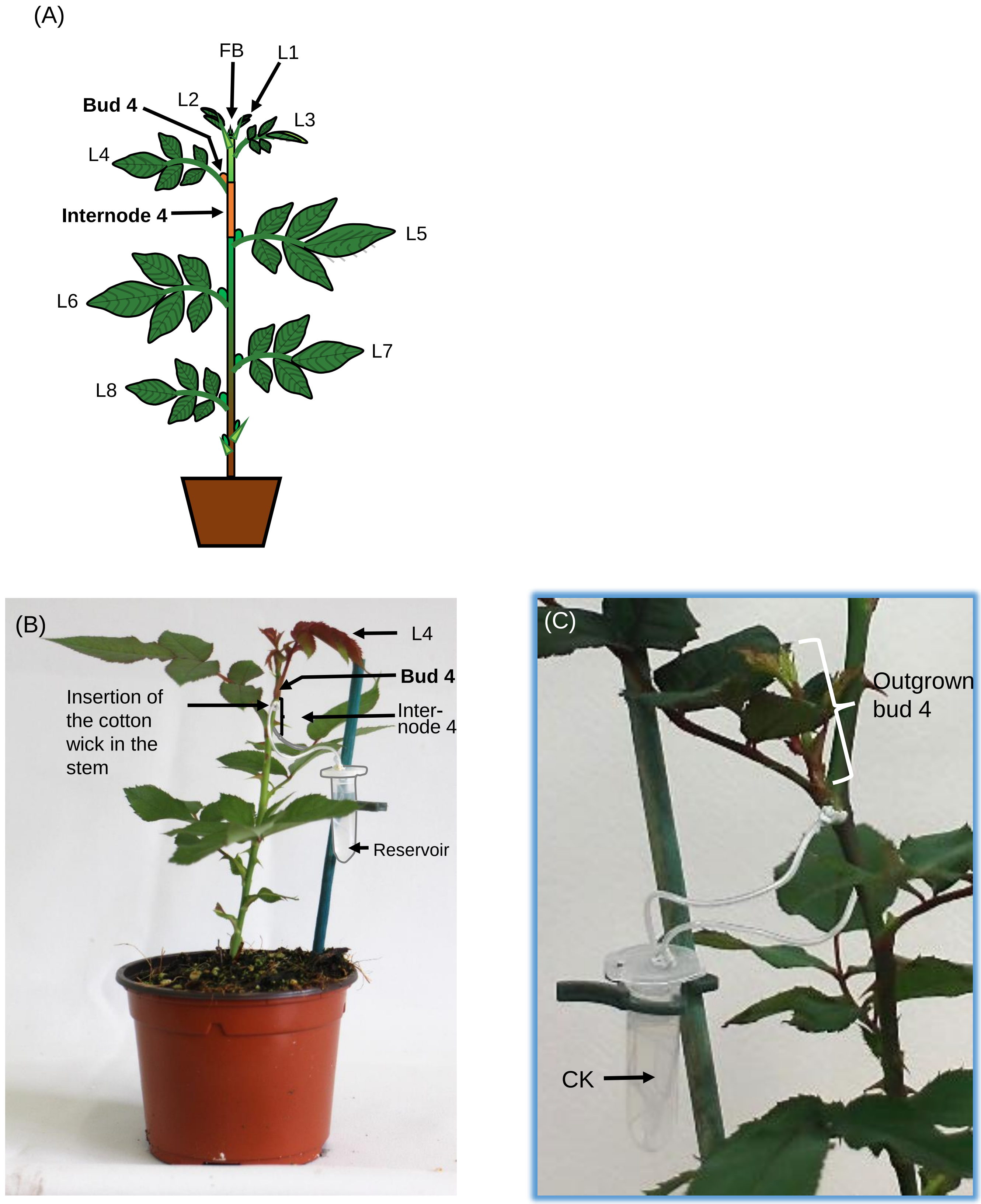

Supplementary materials 2. Schematic representation (A) and picture (B) of a plant at FBV stage. Floral bud (FB) is just visible, leaves (L), buds and internodes are counted basipetaly, bud 4 and internode 4 are shown, on the picture the cotton wick device is represented. (C) Detail of the cotton wick device; with CK supply bud 4 grew out.

Supplementary material 3. Methods for sugar (glucose, fructose, sucrose, starch) quantification

For sugar determination, 17 mg powder was homogenized with 1.3 ml of 80% aqueous ethanol at 80°C for 30 min and then, 700 µl of 50% aqueous ethanol at 80°C for 30 min. The resulting suspension was centrifuged at 5500 rpm for 5 min at 4°C. The supernatant was used for soluble sugar analysis and the pellet for starch analysis. The supernatant was collected and concentrated using a Speed-Vac concentrator, until total ethanol removal, and then diluted in water to a final volume of 600 µl. This volume was used for sucrose, D-glucose and D-fructose content determination using a Konelab 20i sequential automat (Thermo Electron, Vantaa, Finland) and the ENZYTEC™ system (Diagnostics, Viernheim, Germany). The pellet was heated for one night to 60°C and then resuspended with 0.2 ml of 80% aqueous ethanol. The starch in the sample was then hydrolysed as follows. The sample was gelatinized by heat treatment at 100°C for 6 min in presence of 2.9 ml of MOPS (Sigma Aldrich) buffer and 0.1 ml of thermostable α-amylase (3000 $U.ml^{-1}$, Megazyme International Ireland). After cooling to 50°C, the sample was hydrolyzed for 30 min with 4 ml of sodium acetate (200 mM; pH 4.5) and 0.2 ml of amyloglucosidase (200 $U.ml^{-1}$, Mégazyme International Ireland). 2 ml of supernatant was centrifuged at 10000 rpm for 10 min at 4°C. 600 µl was used for D-glucose content determination as described above.

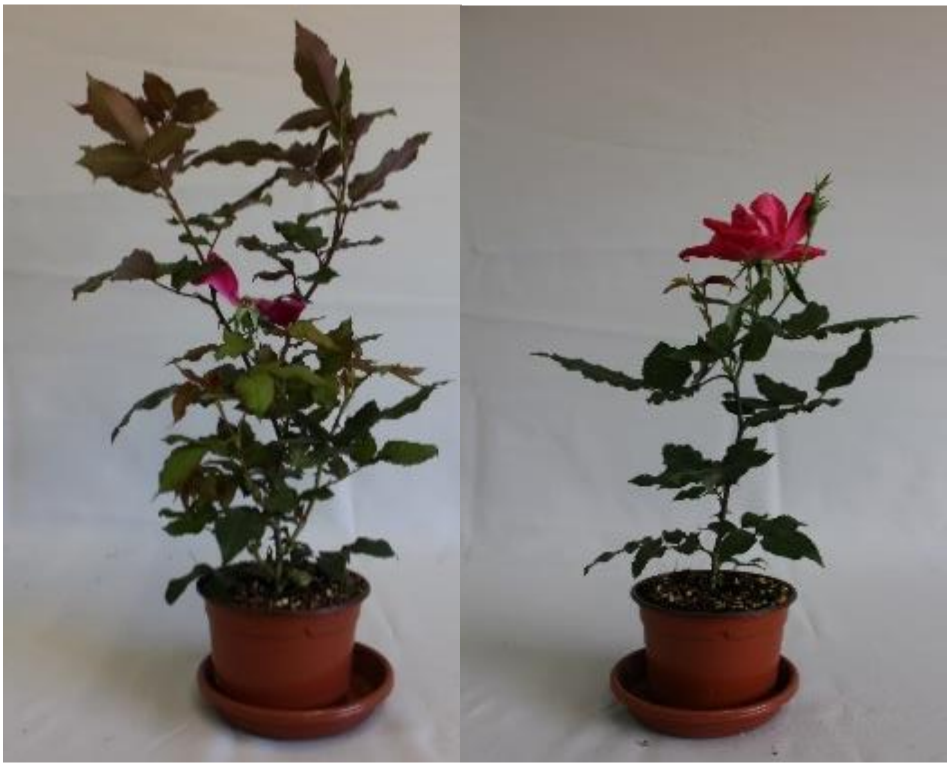

Supplementary Figure S1. Pictures of plants grown under high (left) and low (right) PPFD treatments, 21 days after FBV stage.

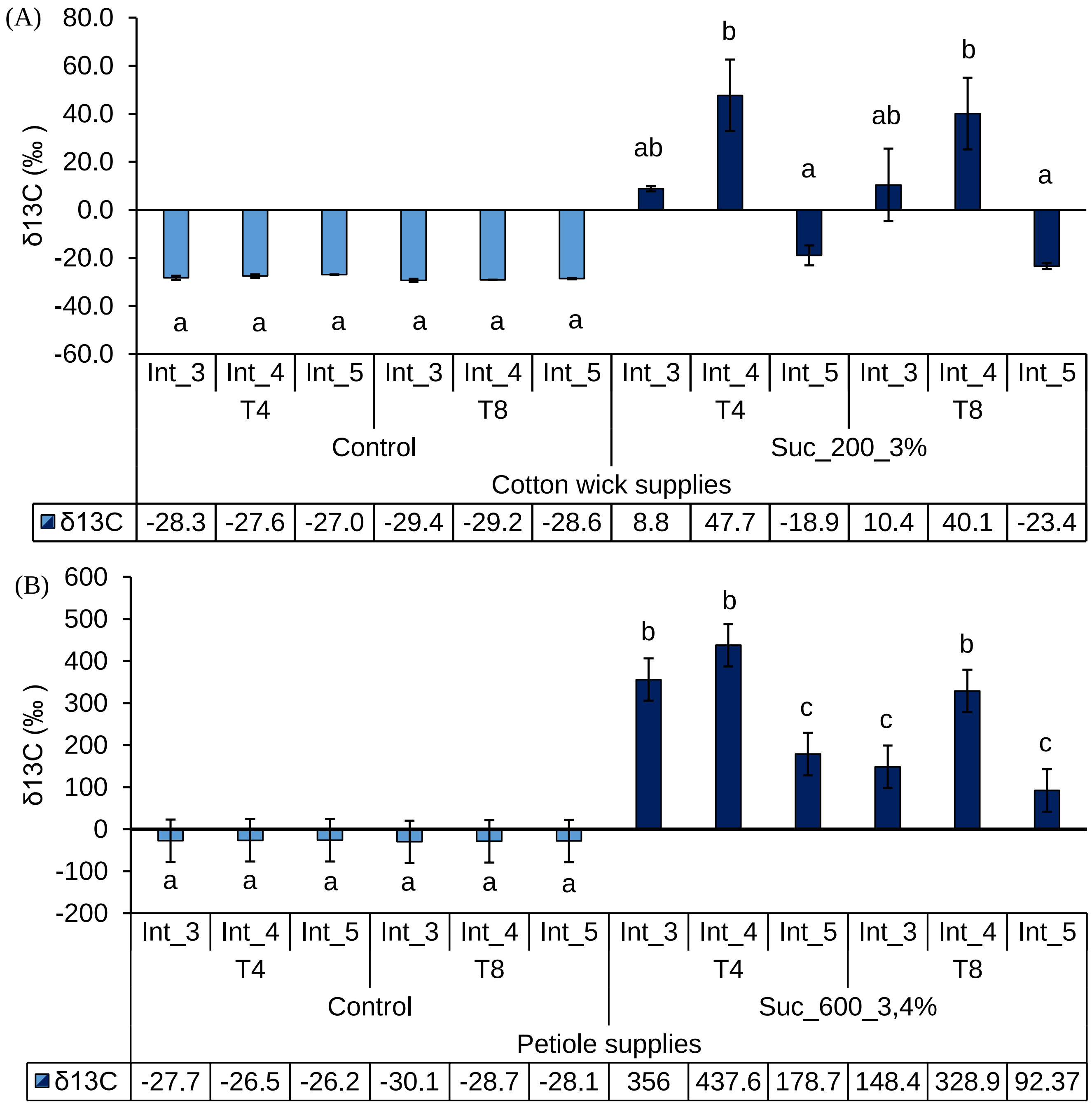

Supplementary Figure S2. Variations in δ^{13}C of internodes 3, 4 and 5 (Int_3, Int_4, Int_5) four days (T4) and eight days (T8) after FBV stage for plants grown under low PPFD and supplied either with labelled sucrose or with water (control). Sucrose or water were supplied through the stem below node 4 at 200mM (A) or through the rachis of leaf 4 at 600 mM (B) from FBV stage onward. Internodes and leaves are numbered basipetally along the stem. Error bars represent the standard error of the mean (SE). Different letters indicate significant differences (ANOVA followed by a Tukey test, P<0.05).

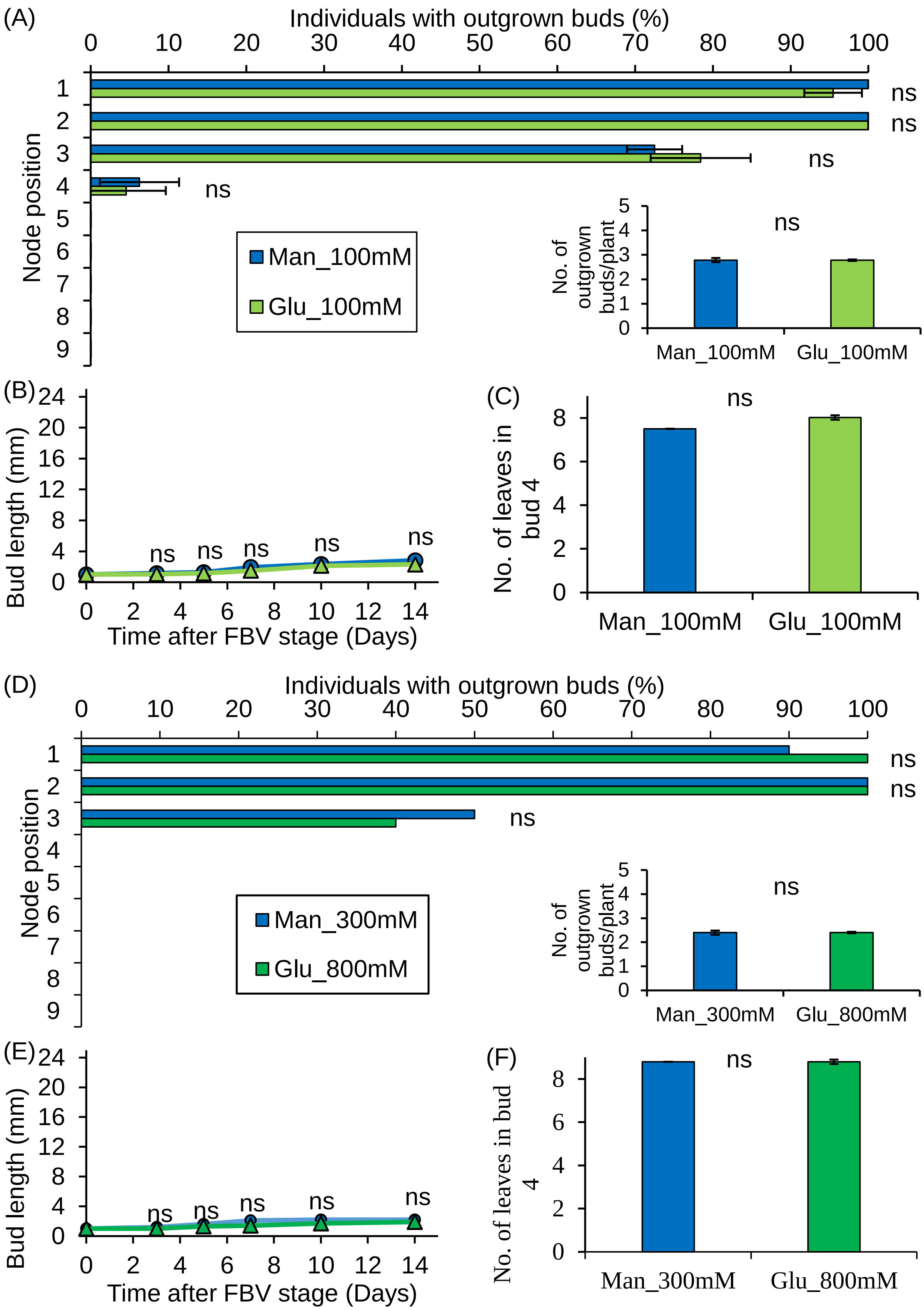

Supplementary Figure S3. Impact of glucose supply on bud outgrowth pattern of plants grown under the low PPFD treatment. Glucose was supplied through the stem below node 4 at 100 mM (A, B, C) or through the rachis of leaf 4 at 800 mM (D, E, F) from FBV stage onward, and mannitol was used as non metabolizable control. (A, D) Effect of glucose supply on the percentage of individuals with outgrown buds 14 days after FBV according to bud position along the shoot. (A-inset, D-inset) Total number of buds per plant that grew out 14 days after FBV. (B, E) Elongation of bud 4 along time. (C, F) Number of leaves in bud 4, 14 days after FBV. Buds are numbered basipetally along the stem. Each point represents the average of two biological repetitions in fig. A, B, C or of 10 plants grown in the same experiment in fig. D, E, F. Error bars represent the standard error of the mean (SE). Different letters indicate significant differences between sugar supplies, ns indicates an absence of significant difference (ANOVA, P<0.05 for all figures, except for D-main graph: Chi-2 test).

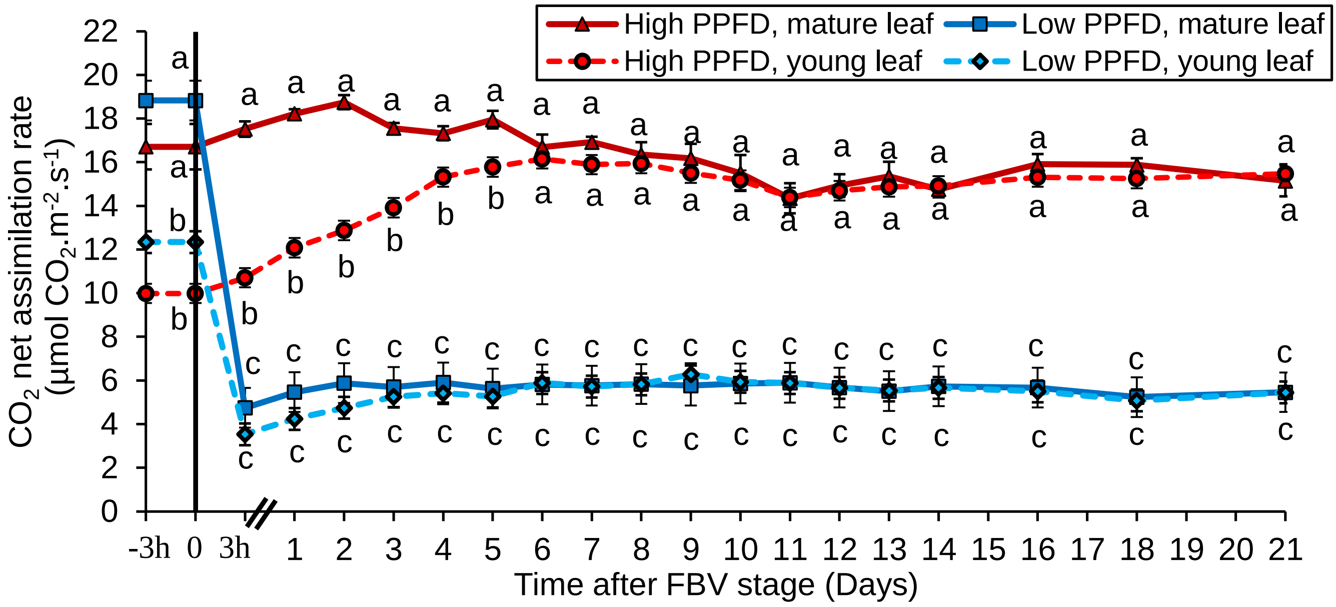

Supplementary Figure S4. Effects of PPFD on CO_2 net assimilation rate of a mature leaf (leaf 6) and a young leaf (leaf 4). Leaf CO_2 net assimilation rate measurements were performed at 540 $\mu mol.m^{-2}.s^{-1}$ before FBV stage, and at 540 or 90 $\mu mol.m^{-2}.s^{-1}$, for plants grown under the high and low PPFD treatments, respectively, from FBV stage onward. Leaves are numbered basipetally along the shoot. Each point represents the average of five plants. Error bars represent standard error of the mean (SE). Different letters indicate significant differences between treatments and leaves within a time point (ANOVA followed by a Tukey test, $P<0.05$).
